# Analysis of Different Methods of Extracting NSAIDs in Biological Fluid Samples for LC-MS/MS Assays: Scoping Review

**DOI:** 10.3390/metabo12080751

**Published:** 2022-08-16

**Authors:** Viviane Silva Siqueira Sandrin, Gabriela Moraes Oliveira, Giovana Maria Weckwerth, Nelson Leonel Del Hierro Polanco, Flávio Augusto Cardoso Faria, Carlos Ferreira Santos, Adriana Maria Calvo

**Affiliations:** Department of Biological Sciences, Bauru School of Dentistry, University of São Paulo, Bauru 17012-901, São Paulo, Brazil

**Keywords:** liquid chromatography, liquid–liquid extraction, mass spectrometry, sample preparation methods

## Abstract

The aim of this study was to carry out a systematic investigation and analysis of different drug extraction methods, specifically non-steroidal anti-inflammatory drugs in biological fluid samples, for Liquid Chromatography in Mass Spectrometry assays (LC-MS/MS). A search was carried out in the main databases between 1999 and 2021, following the Preferred Reporting Items for Systematic reviews and Meta-Analyses extension for Scoping Reviews (PRISMA-ScR) checklist. Data were obtained through PubMed, Lilacs, Embase, Scopus, and Web of Science databases using the Boolean operators AND and OR. Studies were pre-selected by title and abstract by two independent reviewers. The selected texts were read in full, and only those that were complete and compatible with the inclusion and exclusion criteria were eligible for this research. A total of 248 references were obtained in the databases. After removing the duplicates and analyzing the titles and abstracts, 79 references were evaluated and passed to the next phase, which comprised the complete reading of the article. A total of 39 publications were eligible for this study. In 52% of the studies, the authors used the liquid–liquid extraction method (LLE), while in 41%, the solid-phase extraction method (SPE) was used. A total of 5% used microextraction methods and 2% used less-conventional techniques. The literature on the main methods used, the LLE and SPE methods, is extensive and consolidated; however, we found other studies that reported modifications of these traditional techniques, which were equally validated for use in LC-MS/MS. From this review, it is concluded that the diversity of techniques, reliability, and practical information about each analytical method used in this study can be adapted to advances in LC-MS/MS techniques; however, more ecological, economic, and sustainable approaches should be explored in the future.

## 1. Introduction

Non-steroidal anti-inflammatory drugs (NSAIDs), whether prescription or over-the-counter, are typically the class of medications of choice for patients to control inflammatory signs and symptoms. It is estimated that more than 30 billion NSAIDs are sold per year [1]. This class of drugs is generally effective when used to control pain, as its action occurs in the inhibition of cyclooxygenase. However, the use of NSAIDs is also associated with serious adverse reactions, mainly in the gastrointestinal tract, and renal and cardiovascular systems [1,2].

Currently, numerous tests with such drugs have been quantified by the technique of liquid chromatography coupled to mass spectrometry (LC-MS/MS), since the selectivity of this method and the sample preparation are less expensive. These assays, which usually manipulate samples of blood, plasma, saliva, urine, among other biological fluids, are superior when compared to immunoassays or gas chromatography–mass spectrometry (GC-MS) [3,4].

The manipulation or pre-treatment of the sample comprises one of the most time-consuming and laborious steps of the analytical procedures, and for years it was considered of minor importance. The purpose of sample preparation is to remove matrix components that interfere with separation and/or detection and conversion of the analyte to a suitable form and concentration, increasing sensitivity. Currently, with improvements in chromatography and liquid chromatography columns, making the conversion of the analyte to a form suitable for separation is often unnecessary [5].

The peculiar nature of biological samples makes their preparation for LC-MS/MS analysis a challenge in itself. Most clinical samples are aqueous (single matrices); however, not all preparation techniques can be used for water-based samples, or belong to the group that typically includes multiple components that occupy or supply ions, such as hydrogen, sodium, and ammonium, causing ion suppression or enhancement in LC-MS/MS analysis [5,6,7]. Salts and small hydrophilic molecules can have this effect, while compounds affect droplet formation by acting as surfactant compounds in the matrix. They also affect ionization efficiency and result in ion suppression or enhancement. Finally, phospholipids, common in clinical specimens, are well known for their ion-suppressing effect. Therefore, the removal of these compounds is critical for LC-MS/MS analysis. Examples of complex matrices are food, urine, plasma, saliva, and hair, among others [5,6,7].

## 2. Extraction Techniques Found in the Literature for Tests in Liquid Chromatography and Mass Spectrometry

Affordable, effective, and more environmentally friendly preparation techniques have gained space in analytical chemistry and progressed towards the development of new methods aimed at the miniaturization of well-established classical techniques [8]. The means commonly used are divided into two categories, with different extraction phases, namely the methods that use solvents, such as the liquid–liquid extraction (LLE) and dispersive liquid–liquid microextraction (DLLME) methods and their possible modifications, among others, and the extraction methods using sorbent materials, such as solid-phase extraction (SPE), stir bar sorption extraction (SBSE), and solid-phase microextraction (SPME).

### 2.1. Methods That Use Solvents

I. Liquid–Liquid Extraction (LLE)

It is a typical technique used for the preparation of biological samples of an aqueous nature. An equivalent or greater extraction solvent is used to extract all analytes from the original samples. After extraction, the solvent is evaporated and reconstituted. The separation of components depends on the difference in the distribution of the components among immiscible liquids. The feed solution represents a phase and the solvent used to carry out the operation represents the second phase. Mass transfer of the liquid solute occurs from the feed solution to the phase solvent. LLE has a number of disadvantages that restrict its use in laboratories, including limited selectivity, difficulty in automation, and an inability to handle emulsions. The solvents used are normally non-polar organic solvents. When using them, hydrophobic analytes are extracted into the organic layer; however, other non-polar components (e.g., serum lipids) are often co-extracted. LLE is used in the extraction of NSAIDs, such as ibuprofen, nimesulide, piroxicam, and ketorolac, among others [7,9,10,11,12,13,14].

II. Parallel artificial liquid membrane extraction (PALME)

PALME was introduced in 2013 as a new extraction technique. The technique is an extension of liquid-phase microextraction (LPME) in a 96-well format. Two 96-well plates, a donor plate, and a recipient plate, are used to perform the extractions. PALME is performed with commercially available 96-well plates and the extraction procedure offers a simple workflow. Its automation potential is high in addition to offering a high degree of sample cleanliness and can be considered a contribution to “green chemistry”, as the use of organic solvent per sample is low (3–5 μL) [15].

The literature reports a modern perspective for miniaturized LLE, based on the so-called parallel artificial liquid membrane extraction. PALME is performed with flat membranes in a 96-well plate sandwich format. At PALME configuration, target analytes are extracted from a small volume of biological fluid through a flat artificial liquid membrane of an organic solvent immiscible in water and in an aqueous acceptor solution. After PALME, the aqueous acceptor solutions are analyzed directly on LC-MS/MS. In contrast with the single-drop liquid-phase microextraction (SDME) and hollow-fiber liquid-phase microextraction (HF-LPME) techniques, which have been explored for many years without major advances in terms of commercial equipment, the time required for the development of PALME from the present study to its automation will occur in a short space of time. This technique shows promising results in the use of NSAIDs, such as ketoprofen, fenoprofen, flurbiprofen, and ibuprofen [15,16].

III. Magnetic solvent bar liquid-phase microextraction (MSB-LPME)

MSB-LPME bar is a contemporary and alternative extraction method that provides a simple and easy method of extracting analytes from complex matrices. Its devices are cheaply manufactured and easily assembled. The stainless-steel wire is inserted into the hollow-fiber cavity, and the stainless-steel wire is used as a magnetic stirrer, achieving a magnetic separation that is easily isolated from the sample matrix with an external magnetic field. This procedure for the treatment of the sample is uncomplicated and several experimental conditions are being studied and optimized and their performances evaluated.

In addition to immobilizing the extraction solvent, the hollow fiber also has a cleaning ability due to its microporous structure in the membrane wall. However, the filtering effect of hollow fiber is insufficient for the elimination of large molecules (e.g., proteins) in blood samples. NSAIDs such as ketoprofen, naproxen, indomethacin, and diclofenac have already been tested in this new method using human serum as a matrix [17,18].

### 2.2. Methods That Use Sorbents

An alternative technique, and the most used in clinical laboratories, is SPE. The devices consist of small columns that contain cartridges with appropriate packaging, and the choice of the sorbents will depend on the analysis to be extracted. The sorbent is isolated, and a specific organic solvent is used to elute the analyte. Among the advantages of SPE are selectivity, flexibility, and high automation potential. SPE products are available in various shapes, sizes, and separation mechanisms, such as polar, non-polar, ion-exchange, etc. The 96-well plate format is suitable for automation and is typically employed in high-sample-throughput clinical laboratories. It is a common sampling technique in several areas, including pharmaceutical, food, and clinical, among others. Some advantages of SPE are a greater enrichment factor; absence of emulsion, safety with respect to more dangerous samples, low cost, and easy automation. NSAIDs such as etoricoxib, celecoxib, ketoprofen, naproxen, ibuprofen, etc., have already been tested using SPE as an extraction technique in matrices, such as human plasma, urine, and whole blood [10,19,20,21,22,23,24,25].

I. Solid-phase microextraction in-tube online (IN-TUBE SPE)

It is an efficient sample preparation technique that uses an open tubular capillary column as a continuous phase microextraction device (SPME) and can be coupled online with High-Performance Liquid Chromatography (HPLC) or LC-MS/MS [26].

This technique is designed to solve the problems related to the use of conventional SPME fiber, such as the use of conventional fiber, and the low capacity of high-quality special fiber film coatings. From a fiber optic way, a capillary layer coating on the surface of a surface serves as the SPE fabrication means to a capillary layer coating on its inner. IN-TUBE SPE has also been called “treated capillary microextraction”, allowing for direct delivery of analytes into the aqueous phase and a concentration of target analytes in the internal-treated stationary phase in a capillary. The analytes can then be desorbed by introducing a mobile phase stream or using a static desorption solvent when the analytes are more strongly adsorbed to the capillary coating. Compounds can then be injected by LC into the analysis column for analysis. As an alternative to a coated fiber, a capillary is internally coated, through which the sample repeatedly flows or is withdrawn. The main advantage of this technique is that it allows automation of the SPME-HP process, allowing continuous operation, desorption, and injection using a standard autosampler. In addition, it has lower detection limits compared to SPME-HPLC fiber systems [26,27].

The disadvantage would be the need for a lot of cleaning, as the capillary can be easily blocked. To avoid blocking the capillary column and flow lines, it is necessary to select or centrifuge the sample solutions before the withdrawal. Even if yields are low, individuals can be collected in a reproducible way using an autosampler and all of them can be presented in a collection column after a collection. It can be used in matrices, such as human plasma and environmental water, for the extraction of NSAIDs, such as ketoprofen, fenbufen, and ibuprofen [26,27,28].

### 2.3. Scoping Review

Suitable for broad topics, the scoping review allows the gathering of several study designs, which is what distinguishes it from the systematic review, as its objective is not to seek the best evidence about an intervention or experience but rather to gather the various types of evidence and show how they were produced. Additionally, as in primary studies, it is the question that guides the review methodology to be adopted [29,30]. In this way, the scoping review assists reviewers who need to examine emerging evidence and how research is being conducted in already-consolidated areas [29,30].

The aim of this study was to carry out a systematic investigation and analysis of different drug extraction methods, specifically non-steroidal anti-inflammatory drugs in biological fluid samples, for Liquid Chromatography in Mass Spectrometry assays (LC-MS/MS). For this purpose, articles indexed and/or listed on PubMed, Lilacs, Embase, Scopus, and Web of Science databases, between 1999 and 2021, were consulted. Therefore, the following research question was formulated:

- What is the methodology and/or techniques most used for the extraction of non-steroidal anti-inflammatory drugs in bioanalytical assays using high-performance LC-MS/MS?

## 3. Material and Methods

This study followed the Preferred Reporting Items for Systematic reviews and Meta-Analyses extension for Scoping Reviews (PRISMA-ScR) checklist. We registered the final protocol was using Open Science Framework on 24 April 2022 (https://osf.io/nmjpy/?view_only=20246fddd05a41f9b92c536e2726440d) (accessed on 19 July 2022). [30].

We obtained data in PubMed, Lilacs, Embase, Scopus, and Web of Science electronic databases. Our search strategy used Boolean operators (AND and OR): (“Analytic sample preparation methods” OR “Extraction, Liquid–Liquid” OR “Liquid–Liquid Extraction” OR “Extraction, Liquid Phase” OR “Liquid-Phase Microextraction” OR “Solid-phase extraction” OR “Extraction, solid phase” OR “Solid-phase microextraction”) AND (“Anti-Inflammatory Agents, Non-Steroidal” OR “Non-Steroidal Anti-Inflammatory Agents” OR “NSAID” OR “Aspirin-Like Agents”) AND (“Saliv *” OR “Blood” OR “Plasma” OR “Blood plasma” OR “Plasma, blood” OR “Serum” OR “Blood serum”) AND (“Mass Spectrometry” OR “LCMS” OR “LC/MS”). We used End-Note reference manager to save search records and eliminate duplicate references.

I. The selection of studies was carried out by two reviewers independently so that in the first step (pre-selection), titles and abstracts were read and, in the second step, the full texts, to filter only those that were compatible with the eligibility criterion. The following inclusion criteria were applied:
II. Studies with NSAID;
III. Studies with LC-MS/MS;
IV. Studies in English;
V. Studies that presented similar analytical methodology;
VI. Studies covering extraction methods targeting well-established classical techniques and their miniaturization.

Exclusion Criteria:

I. Studies with drugs other than NSAID;
II. Studies with gas, ion-exchange, and affinity chromatography;
III. Studies carried out with animals;
IV. Literature reviews;
V. Not published in English;
VI. Studies that did not allow access to the full content.

When reading the full text, we provided the following data: first author, year and country of origin of the study; type of study, analyte, equipment used for analysis, type of ionization, sample preparation method, and the matrix used. We included articles that contained these data in the review. During all stages of study selection, a third reviewer helped to solve discrepancies.

## 4. Results

### 4.1. Selection of Studies

**Figure 1:**
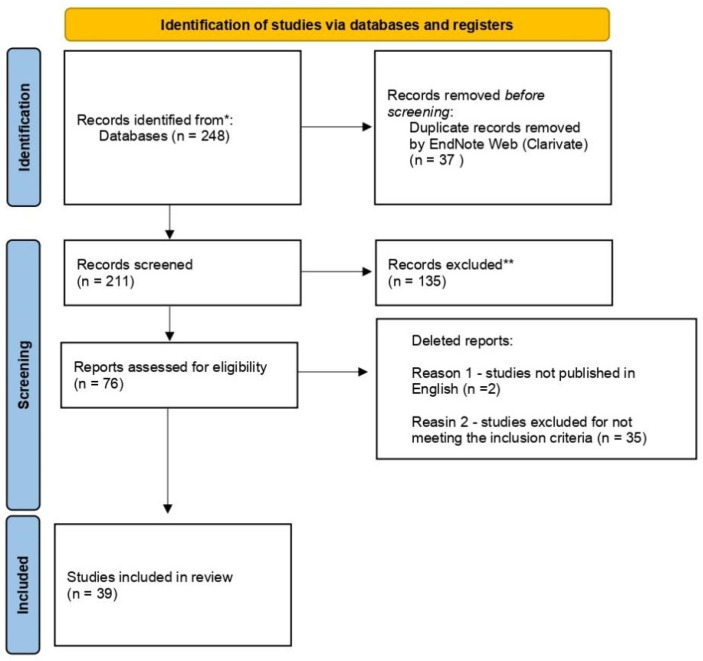
PRISMA 2020 flowchart for new systematic reviews only included database and registry searches [31] (accessed on 19 July 2022).

The search in the databases resulted in 248 studies. After removing the duplicates, 211 remained. After reviewing the titles and abstracts, 79 studies were evaluated and passed to the next phase, which comprised the complete reading of the article. A total of 39 publications were considered eligible for this review (Figure 1).

### 4.2. Study Characteristics

The vast majority of the studies found were bioanalytical trials or bioequivalence studies, published in English from 1999 to 2021, carried out in Norway, Japan, Germany, India, Brazil, United States, Korea, China, United, Kingdom, Macedonia, Egypt, and Australia. A description and classification of the studies found are presented in Table 1.

### 4.3. Summary of Included Studies

**Figure 2:**
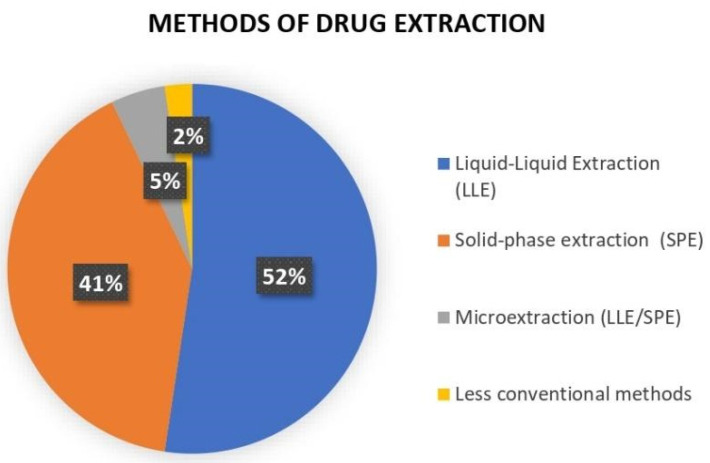
Synthesis of results.

After analyzing 39 well-described studies that met our inclusion and exclusion criteria, two extraction techniques stood out in practical use during bioanalytical assays. In 52% of the included studies, the authors used LLE, while 41% used SPE. Only 7% had used less-conventional methods to carry out their work and only 5% of the researchers used microextraction techniques (Figure 2).

### 4.4. Limitations of the Scoping Review Process

Our scoping review has some limitations. To make this analysis more feasible, we included a random sample of analyses performed on NSAIDs, with diversified matrices. In this sense, our results can only be generalized to studies that focus on non-steroidal anti-inflammatory drugs. Furthermore, this scoping review was a huge undertaking and our results are only up to date until the year 2021.

## 5. Discussion

Sample preparation is one of the indispensable pillars of the science of analytical separation and LLE is among the simplest and most widely used sample preparation techniques. This fact was well observed in our review, as 52% of the studies presented LLE as the technique of choice. LLE is based on the transfer of a solute from an aqueous sample to a water-immiscible solvent, with extraction efficiency determined by the solute distribution coefficient between water and the receiving solvent [57].

According to Bitas et al., LLE is the technique most commonly used in sample preparation and the one that showed the highest selectivity among the simple solvent extraction methods [58]. It is probably the oldest of the techniques applied to determine various chemical compounds [59].

However, even though it has wide use and good analytical performance, LLE has several disadvantages, such as emulsion formation, analyte loss, sample contamination, low sensitivity, automation difficulties, and a need for large sample volumes and organic solvents [58,60,61]. At the present time, LLE is considered an expensive, time-consuming technique and does not meet the current requirements of green analytical chemistry [62].

Despite innovative trends in sample handling that delve into the development of faster, safer, and more environmentally friendly extraction techniques, both LLE and SPE are still useful and widely accepted techniques for the exhaustive extraction of contaminants in organic or biological matrices [63].

This review also highlights that SPE is used as an analytical method in 41% of studies performed for LC-MS/MS assays. SPE is a classic and widely used extraction technique for biofluids and can be applied in manual, semi-automatic, or automatic formats, such as the 96-well Hydrophilic–Lipophilic Balance SPE Block, widely used in bioanalytical assays. Through customized reports and the use of robotic systems, such as the Zymate XP robot, a storage carousel of SPE consumables and final extracts, which allows the construction of a bespoke SPE station, can be obtained [62].

In any case of application, SPE compared with LLE reduces the volumes of organic solvents used, in addition to the possibility of emulsion formation being strongly limited [59]. However, SPE demands an extensive and time-consuming procedure when compared with modern techniques, such as SPME and Micro SPE, which would be a disadvantage, as the innovative (but less used) techniques eliminate the sample pre-treatment steps and analysis time [49,62].

Nevertheless, unfortunately, SPE is relatively expensive, its consumption of organic solvents is considerable, and LC-MS/MS can still be subject to some interference from certain endogenous compounds [64].

In this work, we also discussed how microextraction techniques have advantages over classical techniques (LLE or SPE), such as minimal use of solvents and reduced sample size, in addition to work optimization [26,65]. In our analysis, we found two efficient microextraction techniques that are well employed in LC-MS/MS assays, namely supramolecular magnetic solvent-based liquid-phase microextraction (SUPRAS) of hexafluoroisopropanol (HFIP)-alkanol and online in-tube solid-phase microextraction. These techniques represent 5% of our results.

Liquid-phase microextraction based on supramolecular magnetic solvent (SUPRAS) of hexafluoroisopropanol (HFIP)-alkane consists of immobilizing the extraction solvent. The hollow fiber has a cleaning ability due to the microporous structure in the membrane wall. However, the filtering effect of hollow fiber is not sufficient to eliminate large molecules (e.g., proteins) in blood samples [17].

The presence of large molecular substances in blood samples not only interferes with instrumental analysis but also blocks the hollow-fiber membrane pores, affecting the efficiency of mass transfer in the extraction process, and requiring a pre-treatment step. This step took place through SUPRAS, which is a type of water-immiscible nano/microstructural liquid that originated from the self-assembly of amphiphilic molecular aggregates (micelles or vesicles) induced by specific environmental conditions [66]. In a simple and fast synthesis, the interactivity generated by SUPRAS can improve the extraction efficiency for a wide polarity of analytes. This excellent performance makes SUPRAS based on THF-alkyl carboxylic acid/alkanol widely applied in the pre-treatment of various samples of complex matrices [66,67]. This new extraction method is simple, environmentally friendly, a highly effective, and shows promising application potential in the analysis of blood samples and other complex samples [17].

On the other hand, in-tube Solid-Phase Microextraction, known as in-tube SPME, is an effective sample preparation technique, as it makes use of an open tubular capillary column as an SPME device, and can be coupled online with HPLC or LC-MS/MS [27].

It was developed to overcome problems related to the use of conventional SPME fiber, such as fragility, low sorption capacity, and leakage. Unlike fiber SPME, in-tube SPME typically uses a piece of fused silica capillary with a stationary phase coating on its inner surface (e.g., a small piece of column for gas chromatography) for extraction. In-tube SPME is called “coated capillary microextraction”. This method directly extracts target analytes in aqueous matrices and concentrates the analytes in the stationary phase coated inside a capillary [26,27,68].

The analytes can be desorbed by introducing a mobile phase stream or using a static desorption solvent, with the analytes being more strongly adsorbed to the capillary coating. The desorbed compounds can later be injected into the LC column for analysis. The main advantage of this technique is the possibility of automating the SPME-HPLC process, allowing the extraction, desorption, and injection to be carried out continuously, operating a standard automatic sampler that, when automated, reduces the total analysis time and is more accurate than the manual techniques [26,27,68,69].

The main disadvantage of the technique would be the need for very clean samples, as the capillary is easily blocked. Therefore, to avoid blocking the capillary column and flow lines, it is crucial to filter or centrifuge the sample solutions prior to extraction. Even though yields are usually low, such compounds must be extracted in a reproducible way, using an autosampler, and all extracts can be introduced into an LC column after in-tube SPME [26,27,69].

In our search, we also see a totally new methodology, presented by Ref. [64], for the preparation of biological samples, in particular for the miniaturized LLE in a multi-well plate format, equivalent to 2% of our results [16,64].

In the PALME method, samples are loaded into individual wells in 96-well donor plates. Two 96-well plates, one donor plate and one receiving plate, where the analytes of choice are individually extracted through corresponding liquid membranes, each containing a few microliters of organic solvent and a volume of microliters of aqueous solution, are used to perform the extractions [15,16,64].

Introduced as an innovation in extraction techniques, this is an extension of LPME, which offers a simple workflow with high automation potential. It is considered a contribution to “green chemistry”, as it provides less use of organic solvent per sample, around 3–5 μL, and a working time of 15 to 30 min, in addition to proving to be a valid extraction method for basic hydrophobic drugs from human plasma, allowing combinations with other methods and compatibility with LC-MS/MS [15,16,64].

PALME provided excellent sample cleanliness and is definitely susceptible to future automation and high-throughput operations. Further development of PALME is expected soon but for this to be successful more experimental data are needed. Kristine Skoglund Ask, Elisabeth Leere Øiestad, Stig Pedersen-Bjergaard, and Astrid Gjelstad (2018) hope to find a commercial supplier of 96-well plates with PALME-appropriate polypropylene membranes and definitively automate the process on an existing lab platform. It is also expected that it will be possible to run PALME on plates with more wells, e.g., 384-well plates [15,16,64].

## 6. Conclusions

This review presented and described several methods used by researchers to extract drugs from biological fluid samples, in particular NSAIDs, as an essential step for further analysis in LC-MS/MS.

The literature on the main methods used, the LLE and the SPE method, is extensive and consolidated, but we found other studies that mention a diversity of equally validated techniques for use in LC-MS/MS. From this review, it is concluded that the diversity of techniques, reliability, and practical information about each analytical method used in this study can be adapted to advances in LC techniques, however, more ecological, economic, and sustainable approaches should be explored in the future.

## Figures and Tables

**Table 1:** Systematic investigation and analysis of different drug extraction methods.

Author/Year/Country	Type of Study	Analyte	Equipment	Ionization	Sample Method Preparation	Matrix
Ask, 2018 Norway, [15]	Microsampling Assay	Amitriptyline, quetiapine, ketoprofen, fenoprofen, flurbiprofen and ibuprofen	UHPLC-MS/MS Thermo Scientific LTQ XL Linear Ion Trap (Thermo Scientific, Califónia, USA)	Electrospray (ESI)	Liquid–Liquid Extraction (LLE) Parallel Artificial Liquid Membrane Extraction (PALME)	Whole Blood
Banda, 2016 India, [32]	Bioanalytical Assay	Olsalazine Sodium	UHPLC (Shimadzu, Kyoto, Japan) MS/MS Triple quadrupole API-6500 (MDS Sciex, Ontário, Canada)	Turbo Ion spray	LLE	Human Plasma
Barrientos-Astigarraga, 2001 Brazil, [14]	Bioequivalence Study	Nimesulide	LCMS/MS Micromass Quattro II (Waters Corporation/Micromass Uk Ltd., Manchester, UK)	ESI	LLE	Human Plasma
Bharwad, 2020 India, [33]	Pharmacokinetic Study	Fenoprofen	UHPLC Waters Acquity (Waters Corporation, Massachusetts, USA) MS/MS Quattro Premier XE™ (Waters Micro Mass Technologies, Massachusetts, USA)	ESI	Solid-Phase Extraction (SPE)—Orochem DVB-LP	Human Plasma
Bolani, 2021 Brazil, [13]	Bioanalytical Assay	Piroxicam	LCMS/MS Triple quadrupole Quattro Micro (Waters Corporation/Micromass Uk Ltd., Manchester, UK)	ESI	LLE	Saliva
Bonato, 2003 Brazil, [34]	Enantioselective Analysis	Ibuprofen	HPLC (Shimadzu, Kyoto, Japan) MS/MS Triple quadrupole Quattro Micro (Waters Corporation/Micromass Uk Ltd., Manchester, UK)	ESI	LLE	Human Plasma
Brautigam, 2003 Germany, [20]	Bioanalytical Assay	Etoricoxib	LC Degasser Jasco DG 1580-53 (Gross-Umstadt, Germany) MS/MS Triple quadrupole API 3000 (Applied Biosystems, Langen, Germany)	ESI	SPE—Oasis HLB	Human Plasma
Brêtas, 2016 Brazil, [35]	Bioanalytical Assay	Naproxen and sumatriptan	LC-ESI-MS/MS Waters System (Waters Corporation, Massachusetts, USA) MS/MS Quattro LC—triple quadrupole (Waters Corporation, Massachusetts, USA)	ESI	LLE	Human Plasma
Calvo, 2016 Brazil, [36]	Bioanalytical Assay	Piroxicam and 5′-hidroxypiroxicam	LCMS/MS Triple quadrupole Quatto Micro (Micromass UK Ltd., Manchester, UK)	ESI	LLE	Human Plasma and Saliva
Díonisio, 2020 Brazil, [37]	Bioanalytical Assay	Naproxen	LCMS/MS quadrupole 8040 (Shimadzu, Kyoto, Japan)	ESI	LLE	Saliva
Dongari, 2014 USA, [21]	Bioanalytical Assay	Celecoxib	LC–ESI–TOF–MS HPLC Agilent 1100 Series with a Agilent G1969 TOF/MS System (Agilent, California, EUA)	ESI	SPE—Bond Elute C 18	Human Plasma
Dubey, 2019 India, [38]	Bioanalytical Assay	Celecoxib	LC-10 (Shimadzu, Kyoto, Japan) MS/MS API 3200 (MDS Sciex, Ontario, Canada)	Turbo Ion spray	LLE	Human Plasma
Eichhold, 2000 USA, [22]	Bioanalytical Assay	(R)- and (S)-Ketoprofen	HPLC modular Gilson (Gilson Inc., Wisconsin, USA) MS/MS PerkinElmer API III + (MDS Sciex, Ontario, Canada)	ESI	SPE—Oasis HLB	Human Plasma
Gopinath, 2013 India, [10]	Bioanalytical Assay	Naproxen and Esomeprazole	LCMS/MS Agilent Technologies series 1200 triple quadrupole Agilent 6460 (Agilent Technologies, Germany)	ESI	SPE—Oasis HLB	Human Plasma
Halder, 2019 India, [39]	Bioequivalence Study	Nimesulide and 4-hidroxynimesulide	LCMS/MS API 2000 MS/MS Tandem triple quadrupole (MDS Sciex, Ontario, Canada)	ESI	LLE	Human Plasma
Hoke, 2000 USA, [22]	Bioanalytical Assay	Cetoprofen	LCMS/MS PerkinElmer API III + (MDS Sciex, Ontario, Canada) IMPROVED FLUIDITY LIQUID CHROMATOGRAPHY (pcSFC-MS/MS) Gilson (Gilson Inc., Wisconsin, USA)	Turbo Ion spray/ESI	SPE—Oasis HLB	Human Plasma
Lee, 2006 Korea, [40]	Pharmacokinetic Study	Zaltoprofen	HPLC Waters 2795 MS/MS Triple quadrupole Waters Micromass Quattro Premier (Waters Corporation/Micromass UL Ltd., Watford, UK)	ESI	LLE	Human Plasma
Lee, 2008, [41] Korea	Bioanalytical Assay	Etodolac	HPLC Waters 2795 MS/MS Triple quadrupole Waters Micromass Quattro Premier (Waters Corporation/Micromass Uk Ltd., Watford, UK)	ESI	LLE	Human Plasma
Lee, 2014 Korea, [42]	Bioanalytical Assay	Flurbiprofen	HPLC Agilent 1200 series (Agilent Technologies Inc., California, USA) MS/MS API 3200 (MDS Sciex, Ontario, Canada)	ESI	LLE	Human Plasma
Li, 2020 China, [17]	Bioanalytical Assay	Cetoprofen, naproxen, indomethacin, and diclofenac	HPLC 20A (Shimadzu, Kyoto, Japan) MS/MS Triple quadrupole 4000 QTrap (AB Sciex, Washington, USA)	ESI	Liquid-phase microextraction based on supramolecular magnetic solvent HFIP-alkanol with solvent bar (MSB-LPME based on HFIP-alkanol SUPRAS)	Human Serum
Mahadik, 2012 India, [43]	Bioanalytical Assay	Mefenamic Acid	LCMS/MS PerkinElmer API-3000 (MDS Sciex, EUA) coupled to high performance liquid chromatography (Shimadzu, Kyoto, Japão)	Atmospheric Pressure Chemical Ionization (APCI)	LLE	Human Plasma
Mohammed, 2013, United Kingdom, [14]	Microsampling Assay	Ketorolac	HPLC-MS/MS TSQ Quantum Discovery Max triple quadripole (Thermo Scientific, USA)	ESI	LLE	Human Plasma
Nakov, 2015 Macedonia, [23]	Bioanalytical Assay	Ibuprofen	HPLC-MS/MS TSQ Quantum Discovery Max triple quadripole (Thermo Scientific, USA)	ESI	LLE/SPE	Human Plasma
Nakov, 2016 Macedonia, [44]	Bioanalytical Assay	Ibuprofen	HPLC-MS/MS TSQ Quantum Discovery Max triple quadripole (Thermo Scientific, USA)	ESI	LLE	Human Plasma
Ojha, 2009 India, [45]	Bioanalytical Method Validation	4-methylaminoantipyrine—dipyrone active metabolite	LC—Atmospheric pressure ionization (Ion Spray) MS Simple Quadrupole (PerkinElmer MDS Sciex, USA)	APCI	LLE	Human Plasma
Park, 2012, [46] Korea	Bioanalytical Assay	Celecoxib	HPLC Agilent 1100 (Agilent, USA) MS/MS Triple quadrupole API-2000 (MDS Sciex, Ontario, Canada)	ESI	LLE	Human Plasma
Patel, 2008 India, [47]	Bioanalytical Assay	6-methoxy-2-naphthylacetic acid - nabumetone active metabolite	LCMS/MS Triple quadrupole API-3000 (Shimadzu, Kyoto, Japan)	Turbo Ion spray	SPE—Oasis HLB Cartridges	Human Plasma
Patel, 2012 India, [24]	Bioanalytical Assay	Sumatriptan and naproxen	UPLC Waters Acquity System and a triple quadrupole Waters Quattro Premier XE (Waters Corporation, Massachusetts, USA)	ESI	SPE —Phenomenex Strata-X Cartridges	Human Plasma
Patel, 2013 India, [48]	Bioanalytical Assay	Diflunisal—salicylic acid difluorophenyl derivative	LCMS/MS Triple quadrupole API-3000 (Shimadzu, Kyoto, Japan)	ESI	SPE—Oasis HLB Cartridges	Human Plasma
Scott, 1999 United Kingdom, [49]	Bioanalytical Assay	Green ford-ware cocktail (Diclofenac)	LCMS/MS 200 series triple quadrupole API-365 (PerkinElmer MDS Sciex, Onario, Canada)	Turbo Ion spray/ESI	96-extraction-well HLB SPE block—automated extraction	Human Plasma and Urine
Shinde, 2012 Korea, [50]	Bioanalytical Assay	Aspirine	HPLC Agilent 1200 series (Applied Biosystems, California, USA) MS/MS QTrap 5500 (Applied Biosystems, California, USA)	ESI	SPE—Discovery DSC-C8 cartridges	Human Plasma
Shirako, 2013, Japan, [51]	Bioanalytical Assay	Ampiroxicam, tenoxicam, piroxicam, meloxicam, and lornoxicam	LCMS/MS API-4000 (AB Sciex, Massachusetts, USA)	ESI	MAX-SPE—Oasis cartridges column	Human Plasma
Suenami, 2006 Japan, [52]	Bioanalytical Assay	Acetaminofen, aspirine, loxoprofen, cetoprofen, acemetacin, oxaprozin, fenoprofen, flurbiprofen, indomethacin, diclofenac, ibuprofen, henylbutazone, flufenamic acid, mefenamic acid, tolfenamic acid, and naproxen	HPLC Alliance 2690 coupled to MS/MS Quadrupole Micromass ZMD (Waters Corporation, Massachusetts, USA)	ESI	SPE—Oasis HLB cartridges	Human Plasma
Sultan, 2005 Egypt, [25]	Bioanalytical Assay	Salicin, salicylic acid, tenoxicam, ketorolac, piroxicam, tolmetin, naproxen, flurbiprofen, diclofenac, and ibuprofen	HPLC 616 model (Waters Corporation, Massachusetts, USA) coupled to a MS/MS Finnigan-MAT TSQ triple quadrupole (Thermo Finnigan, California, USA)	APCI	LLE/SPE—copolymer-based cartridges (poli(N-vinilimidazol-co-divinilbenzeno))	Human Plasma and Whole Blood
Sun, 2016 China, [53]	Bioanalytical Assay	Nimesulide	LC (Shimadzu, Kyoto, Japan) coupled to a MS/MS QTrap5500 (Applied Biosystems, California, USA)	ESI	LLE	Human Plasma
Taylor, 1998 Australia, [54]	Bioanalytical Assay	Indometacin	HPLC (Waters Corporation, Massachusetts, USA) coupled to a MS/MS quadruple API III (PerkinElmer MDS Sciex, Ontario, Canada)	ESI	SPE	Human Plasma
Werner, 2002 Germany, [55]	Bioanalytical Assay	Celecoxib	HPLC (Jasco, Groß-Umstadt, Germany) MS/MS Trap Finnigan MAT LCQ (Thermoquest, Egelsbach, Germany)	APCI	LLE	Human Plasma
Yu, 2012 China, [28]	Bioanalytical Assay	Cetoprofen, fenbufen, and ibuprofen	LC-MS-2010EV HPLC-ESI/MS (Shimadzu, Kioto, Japan)	ESI	In-tube solid-phase microextraction (IN-TUBE SPE)	Human Plasma and Environmental Water
Zhang, 2003 USA, [56]	Bioanalytical Assay	Valdecoxib	HPLC Agilent 1050 (Agilent, California, USA) MS/MS Quadrupole PerkinElmer Sciex API-III-Plus (Ontario, Canada)	ESI	Autmated system SPE RapidTrace™—Bond Elut cartridges	Human Plasma

## Data Availability

Our data are available at this access link https://osf.io/nmjpy/?view_only=20246fddd05a41f9b92c536e2726440d (accessed on 19 July 2022).
